# Low intakes of dietary fiber and magnesium are associated with insulin resistance and hyperandrogenism in polycystic ovary syndrome: A cohort study

**DOI:** 10.1002/fsn3.977

**Published:** 2019-02-27

**Authors:** Dylan A. Cutler, Sheila M. Pride, Anthony P. Cheung

**Affiliations:** ^1^ Department of Obstetrics and Gynaecology University of British Columbia Vancouver British Columbia Canada; ^2^ Grace Fertility & Reproductive Medicine Vancouver British Columbia Canada

**Keywords:** diet, hyperandrogenism, insulin resistance, lifestyle, obesity, PCOS

## Abstract

**Background:**

Women with polycystic ovary syndrome (PCOS) often have insulin resistance (IR) which may be worsened by obesity. The roles of dietary intake and activity are unclear. Our objectives were to determine whether (a) high caloric intake or inactivity explains obesity in PCOS, and (b) dietary composition is associated with PCOS phenotypes.

**Methods:**

Eighty‐seven women with PCOS and 50 women without PCOS participated in this cohort study at a reproductive medicine center. Data collected included 3‐day food and physical activity records, anthropometrics, and metabolic and hormonal assays.

**Results:**

Women with PCOS had increased body mass index (BMI) but similar caloric intake and activity to women without PCOS. There were no differences in protein, carbohydrates, fat, or glycemic load consumption, but women with PCOS consumed less fiber (medians: 19.6 vs. 24.7 g) and less magnesium (medians: 238.9 vs. 273.9 mg). In women with PCOS, those with IR consumed less fiber, less magnesium, and greater glycemic load than those without IR (medians: 18.2 vs. 22.1 g, 208.4 vs. 264.5 mg, 89.6 vs. 83.5). Fiber intake of women with PCOS was negatively correlated with IR, fasting insulin, glucose tolerance, testosterone, and dehydroepiandrosterone sulfate. Magnesium intake was negatively correlated with IR, C‐reactive protein, and testosterone, but positively correlated with HDL cholesterol. Fiber intake and BMI accounted for 54.0% of the variance observed in IR.

**Conclusions:**

Obesity in women with PCOS could not be explained by overeating or inactivity. Increasing dietary fiber and magnesium intakes may assist in reducing IR and hyperandrogenemia in women with PCOS.

## 1 BACKGROUND

Polycystic ovary syndrome (PCOS) is a multisystem disorder reported in 6%–18% of women worldwide and can affect reproductive, metabolic, and mental health (Fauser et al., 2012; March et al., 2010). According to the Rotterdam criteria, the diagnosis of PCOS requires the presence of any two of the following: oligo‐ovulation or anovulation, hyperandrogenism (biochemical or clinical), and/or polycystic ovarian (PCO) morphology on ultrasound after excluding other endocrinopathies (The Rotterdam ESHRE/ASRM‐Sponsored PCOS Consensus Workshop Group, 2004). Women with PCOS often have insulin resistance (IR), dyslipidemia, and obesity. An increased body mass index (BMI) or excess adiposity, particularly visceral abdominal fat, can independently contribute to menstrual irregularities, anovulation, and metabolic abnormalities (Dumesic et al., 2016; Fauser et al., 2012; Lim, Davies, Norman, & Moran, 2012). Moreover, hyperandrogenic phenotypes of PCOS may be at greater risk of developing metabolic syndrome (Shroff, Syrop, Davis, Van Voorhis, & Dokras, 2007).

A review by Lin and Lujan noted large discrepancies in the current research assessing the lifestyles of women with PCOS (Lin & Lujan, 2014). Some studies have reported that women with PCOS consume a higher caloric intake compared to women without PCOS, while others showed no significant differences in either dietary intake or physical activity (Douglas et al., 2006; Graff, Mário, Alves, & Spritzer, 2013; Moran et al., 2013; Wright, Zborowski, Talbott, McHugh‐Pemu, & Youk, 2004; Zhang et al., 2015). While a caloric surplus (excess calories and/or inactivity) is a common cause of obesity, previous studies have indicated that women with PCOS may have a slower basal metabolic rate and/or reduced postprandial thermogenesis (the rate at which food is broken down; Georgopoulos et al., 2009; Robinson et al., 1992). In addition, inconsistent findings have been reported in studies assessing the intakes of fiber and glycemic load in women with PCOS, two important dietary components for managing weight, insulin levels, and dyslipidemia (Barr, Hart, Reeves, Sharp, & Jeanes, 2011; Graff et al., 2013; Moran et al., 2013; Wild, Painter, Coulson, Carruth, & Ranney, 1985). Fiber consumption has been shown to increase satiety, reduce glucose, and decrease both total and LDL cholesterol levels (Weickert & Pfeiffer, 2008). Reducing glycemic load has been shown to assist in weight management, improve glycemic control in diabetics, reduce dyslipidemia, and increase HDL cholesterol levels (Jenkins et al., 2002). One study provided evidence that when 29 women with PCOS followed a low‐glycemic diet for 12 months, they showed a significant increase in sensitivity to insulin, improved menstrual regularity, and improved quality of life scores in comparison to the 21 women with PCOS that consumed a healthy diet of normal glycemic load (Marsh, Steinbeck, Atkinson, Petocz, & Brand‐Miller, 2010).

Specific micronutrients have been discussed in regards to managing PCOS. For example, magnesium status has been inversely associated with metabolic syndrome and BMI in the general population but its role in PCOS is unclear. When magnesium intake was evaluated in women with PCOS, it was found that their intake was not different than women without PCOS and did not correlate with fasting insulin or insulin‐to‐glucose ratio (Douglas et al., (2006)). However, more recent evidence concluded that micronutrient intake, such as magnesium, iron, phosphorus, retinol, and vitamin E, was greater for women with PCOS (Moran et al., 2013). Therefore, it is uncertain if specific dietary micronutrient intakes differ or contribute to higher rates of obesity, insulin resistance, and dyslipidemia in PCOS.

In clinical practice, women with PCOS are often frustrated that, despite lifestyle modification, they have difficulty controlling weight and improving IR. Further, reduced fiber intake and increased glycemic load have been associated with an increased prevalence of type 2 diabetes and metabolic syndrome (Salmeron et al., 1997). We therefore hypothesized that these dietary factors may also be linked to IR in PCOS.

Our main objective was to investigate overall caloric intake, physical activity, and obesity in women with and without PCOS. Our second objective was to assess intakes of main dietary components (protein, carbohydrate, fat, fiber, and glycemic load) and micronutrients to identify associations between these dietary components and obesity, IR, and hyperandrogenism in women with PCOS.

## 2 MATERIALS AND METHODS

### 2.1 Study design, setting, and participants

This observational, cohort study took place from May 2014 to December 2016. Women between the ages 20 and 44 years were recruited at Grace Fertility and Reproductive Medicine in Vancouver, Canada (*n* = 137) at their initial consultation with a reproductive endocrinologist. There were 87 women with PCOS, according to the Rotterdam criteria, and 50 subfertile women without PCOS for comparison. Of the 87 women with PCOS, 49 were categorized as “Hyperandrogenic PCOS (HA)” when all three Rotterdam criteria were present or when hyperandrogenism and oligo‐/anovulation were present without PCO on ultrasound. Hyperandrogenism was defined as the presence of biochemical and/or clinical androgen excess. Biochemical androgen excess was identified if at least one of the following three androgens were elevated according to local laboratory references (total testosterone ≥1.8 nmol/L, androstenedione ≥7.48 nmol/L, or dehydroepiandrosterone sulfate ≥10.8 umol/L). Clinical androgen excess was assessed, depending on ethnicity, by a self‐reported modified Ferriman–Gallwey score. For those of European, Aboriginal, and South American descent, a score of 8 or higher constituted hyperandrogenism while a score of 6 or higher for East Asian participants, and 10 or higher for South Asian participants sufficed (Afifi et al., 2017; Karimah & Hestiantoro, 2017; Wijeyaratne, Balen, Barth, & Belchetz, 2002). The remaining 38 women, with both oligo‐/anovulation and PCO on ultrasound, were categorized as “non‐hyperandrogenic PCOS (Non‐HA).” “PCO on ultrasound” was defined by the Rotterdam criteria as (a) having 12 or more follicles measuring 2–9 mm in diameter in one or both ovaries, or (b) the ovarian volume exceeding 10 cm^3^. It should be noted that many of our participants had >12 follicles, some with 25 or more follicles per ovary which was proposed recently as a criterion for PCO morphology (Dewailly et al., 2014). Women recently diagnosed with or still recovering from an eating disorder (such as anorexia or bulimia nervosa) were excluded.

### 2.2 Dietary and activity assessment

Participants completed a 3‐day food and activity record, which consisted of two weekdays and one weekend day (Stumbo, 2008; Willett, 2012; Yang et al., 2010). They were instructed to provide detailed accounts of their daily food and drink intake. This included the amount (using measuring utensils, scales, or food labels), brand names, flavors, condiments, cooking methods, and time of eating. Participants were provided with a list of common objects to compare to their food portions, if food could not be measured (i.e., dining out). For example, participants were instructed to record that they ate either “¾ cup brown rice, cooked” or its equivalent being a “tennis ball size portion of brown rice, cooked.” Food records were accompanied by electronic photographs of meals. Once food records were returned to the researchers, food items, quantities, details, and commonly forgotten items were verified with each participant. Pedometers (SM‐2000 Step Pedometer by Heart Rate Monitors USA) were provided to quantify participants’ daily steps, in addition to their physical activity record. Participants were encouraged to maintain their typical dietary and activity regimens for the duration of the study.

Dietary intake, comprising total energy, protein, carbohydrates, fat, glycemic load, fiber, and ten micronutrients (including vitamin A, vitamin C, vitamin D, folate, calcium, iron, sodium, zinc, magnesium, and cholesterol), was determined using a nutrition and fitness software program designed for research and clinical purposes (ESHA Food Processor 10.12, 2013). This software accesses data on nutritional components, including calories, protein, carbohydrates, fat, fiber, and micronutrients from Health Canada's Canadian Nutrient File. Every food item from each participant's food record was assessed by trained researchers and manually matched to its equivalent food item in Health Canada's Canadian Nutrient File. To calculate glycemic load, the glycemic index for each food containing carbohydrates was identified based on published literature and manually input into our software (Willett, 2012).

### 2.3 Anthropometrics

Participants’ height, weight, waist and hip circumference, blood pressure, and heart rate were recorded. Body mass index (BMI) and waist‐to‐hip ratio (WHR) were calculated.

### 2.4 Biochemical assays

All participants had a baseline transvaginal ultrasound assessment by the same physician using the EC9‐5/10 endovaginal transducer (SonixTouch, Ultrasonix) and hormone measurements for follicle‐stimulating hormone (FSH), estradiol (E_2_), prolactin (PRL), and thyroid‐stimulating hormone (TSH; Abbott Architect Immunoassay). Women with PCOS had additional hormone and metabolic measurements which included the following: luteinizing hormone (LH), progesterone (P), androstenedione (A_4_), and 17‐hydroxyprogesterone (17‐OHP; Agilent 6410, LCMS methodology); testosterone (T), dehydroepiandrosterone sulfate (DHEAS), and fasting insulin (Roche Cobas e602 Immunoassay); blood glucose levels after a 12‐hr fast (FBG) and at 2 hr after a 75 g oral glucose tolerance test (2 hr glucose level; Roche Cobas c701 Roche Diagnostics, hexokinase/G6P‐DH method); total cholesterol (TC), high‐density lipoprotein cholesterol (HDL‐C), and triglycerides (TG; Roche Cobas c701 Roche Diagnostics enzymatic colorimetric method, and with polyethylene glycol modified enzymes and dextran sulfate for HDL‐C).

The homeostasis model assessment of insulin resistance (HOMA‐IR) was calculated using the formula: HOMA‐IR = fasting blood glucose (mmol/L) × fasting insulin (µIU/ml)/22.5 (Matthews et al., 1985). HOMA‐IR is a reliable clinical tool for measuring insulin sensitivity with a strong correlation to the more laborious glucose clamp measurements (Bonora et al., 2000; Lansang, Williams, & Carroll, 2001). Insulin resistance was defined as a HOMA‐IR ≥3.8, as used in previous studies evaluating women with PCOS (Kar, 2013).

### 2.5 Statistical analyses

Since nutrient intakes can be affected by total food quantity consumed, crude nutrient intakes for fiber and glycemic load were adjusted for total energy intake using the residual method (Willett, Howe, & Kushi, 1997). Protein, carbohydrates, and fats were recorded as percentages of total energy intake, and therefore, were already adjusted for total caloric intake. Dietary under‐reporters were identified according to the Goldberg cutoff method, and analysis was conducted with and without the data of the under‐reporters (Black, 2000). The Goldberg cutoff method establishes an approximate basal metabolic rate based on each participant's height, weight, sex, and age. This value represents the minimal caloric intake necessary to survive, and therefore, any participants with a caloric intake below this value are identified as under‐reporters. The statistical significance found in our analysis was unchanged when under‐reporters were removed, so they were included in all reported results (Black, 2000; Schofield, 1985).

Baseline characteristics and outcome parameters were assessed for statistical normality and compared with either a 2‐sample *t* test or its nonparametric equivalent, Mann–Whitney *U* test for two groups; or, one‐way ANOVA (with post hoc pairwise comparison according to Tukey) or its nonparametric equivalent, Kruskal–Wallis test when groups were stratified by BMI or fiber intake. Relationships between continuous variables were determined by Pearson and Spearman's Rank correlations, as appropriate. Stepwise multiple linear regression was used to identify independent predictors of HOMA‐IR. Analyses were executed using R software. A *p*‐value of <0.05 was considered statistically significant.

## 3 RESULTS

### 3.1 All participants

The ethnicity of study participants was 42% East Asian, 40% European, 15% South Asian, 2% Aboriginal, and 1% South American. Women with PCOS had significantly higher BMI (*p* < 0.001), higher WHR (*p* < 0.01) and were younger (*p* < 0.001) than women without PCOS (Table 1). No significant differences were found between the caloric intake and physical activity levels in women with or without PCOS (Table 1). Macronutrient composition of dietary intake as percentages of protein, carbohydrate, and fat did not differ, nor did the crude or adjusted glycemic loads of the two groups. However, women with PCOS consumed significantly less fiber than women without PCOS (crude: *p* < 0.01, adjusted: *p* < 0.01, Table 1). In addition, women with PCOS consumed less magnesium (crude: *p* = 0.02, adjusted: *p < *0.05) and less vitamin A (crude: *p* = 0.01, adjusted: *p = *0.005) than women without PCOS (Table 1).

**Table 1:** Characteristics and daily dietary intake in women with and without PCOS

	PCOS (*n* = 87)	Non‐PCOS (*n* = 50)
Characteristics		
Age (years)	30.7 (4.6)	35.7 (5.2)^a^
BMI (kg/m^2^)	29.0 (7.1)	24.1 (5.1)^a^
WHR	0.84 (0.08)	0.79 (0.07)^b^
FSH (IU/L)	5.4 (1.7)	6.2 (2.9)
E_2_ (pmol/L)	165.9 (70.5)	201.9 (176.9)
PRL (μg/L)	12.1 (8.7)	10.6 (4.8)
TSH (mIU/L)	2.4 (2.3)	2.0 (0.9)
Dietary intake		
Energy (kcal)	1,783 (1,516–1,966)	1,815 (1,578–2,083)
Step count	6,554 (4,918−9,173)	7,234 (5,558−8,663)
Protein (%)	16.8 (14.2−19.8)	16.4 (14.4−18.8)
Carbohydrate (%)	46.2 (42.4−50.8)	49.0 (42.7−52.2)
Fat (%)	36.0 (32.3−39.2)	34.0 (30.1−38.7)
Fiber (g)		
Crude	19.6 (15.9−23.9)	23.3 (19.4−31.8)^b^
Adjusted	19.6 (15.7−24.0)	24.7 (19.7−30.7)^b^
Glycemic load		
Crude	84.1 (58.9−106.2)	86.0 (68.2−105.1)
Adjusted	83.7 (66.8−105.7)	83.0 (69.0−107.5)
Vitamin A (IU)		
Crude	6,738.0 (2,177.7−10,139.3)	8,591.7 (5,002.5−14,214.9)^b^
Adjusted	6,245.3 (2,533.1−9,401.8)	8,366.1 (4,940.7−14,954.4)^b^
Vitamin C (mg)		
Crude	96.5 (60.5−126.4)	112.8 (60.5−181.7)
Adjusted	100.8 (52.9−176.0)	111.2 (66.7−167.4)
Vitamin D (mcg)		
Crude	2.53 (1.40−4.72)	2.21 (1.31−3.67)
Adjusted	2.83 (1.64−4.69)	2.17 (1.29−3.80)
Folate (mcg)		
Crude	256.5 (168.9−369.9)	283.5 (204.2−355.9)
Adjusted	255.6 (173.4−358.3)	276.1 (211.6−353.9)
Calcium (mg)		
Crude	606.5 (428.2−807.8)	637.5 (465.8−797.7)
Adjusted	658.3 (474.1−799.2)	606.9 (504.6−791.0)
Iron (mg)		
Crude	11.6 (9.0−14.7)	13.3 (11.4−15.0)^c^
Adjusted	11.8 (9.4−14.0)	12.2 (10.7−14.9)
Sodium (mg)		
Crude	2,188.0 (1,517.6−2,874.5)	2,182.1 (1,718.4−2,898.7)
Adjusted	2,242.2 (1,632.5−2,825.1)	2,065.1 (1,601.3−2,873.6)
Zinc (mg)		
Crude	7.85 (5.7−10.6)	8.7 (6.6−10.8)
Adjusted	8.12 (6.48−10.5)	9.10 (7.15−10.1)
Magnesium (mg)		
Crude	236.7 (157.9−326.9)	292.7 (209.6−417.5)^c^
Adjusted	238.9 (185.3−312.6)	273.9 (213.7−402.9)^c^
Cholesterol (mg)		
Crude	284.1 (179.9−430.3)	260.8 (146.0−340.6)
Adjusted	297.5 (183.9−439.8)	254.9 (146.0−338.6)

Note

Statistical significance where “a” denotes *p* ≤ 0.001, “b” denotes *p* ≤ 0.01, and “c” denotes *p* < 0.05. Values are expressed as mean (*SD*) or median (interquartile range). While glycemic load and glycemic index are related, glycemic load accounts for both the amount and quality of carbohydrate while glycemic index refers only to the quality. Fiber, glycemic load and all micronutrients are presented as both raw (crude) and adjusted data. The adjusted amount accounts for overall energy intake using the residual method (Willett et al., 1997).

### 3.2 Participants with PCOS

The characteristics of women with PCOS were summarized and compared according to BMI grouping (normal weight, overweight, and obese) and IR (HOMA‐IR <3.8 vs. HOMA‐IR ≥3.8; Table 2). There was a strong correlation between BMI and HOMA‐IR (*ρ* = 0.72, *p* < 0.001), but no correlation between age and BMI nor HOMA‐IR in our PCOS cohort.

**Table 2:** Characteristics and daily dietary intake of women with PCOS by BMI and insulin resistance

	BMI category			Insulin resistance	
	Normal (*n* = 25)	Overweight (*n* = 31)	Obese (*n* = 31)	HOMA‐IR <3.8 (*n* = 30)	HOMA‐IR ≥3.8 (*n* = 42)
Characteristics					
BMI (kg/m^2^)	22.2 (1.8)	27.0 (1.4)	36.6 (6.3)^a^	26.0 (4.4)	32.7 (7.6)^a^
WHR	0.81 (0.08)	0.85 (0.05)	0.86 (0.09)^c^	0.83 (0.1)	0.86 (0.1)
FBG (mmol/L)	4.9 (0.4)	5.1 (0.6)	5.4 (0.7)^b^	4.8 (0.4)	5.4 (0.7)^a^
2 hr gluc (mmol/L)	5.3 (1.6)	6.6 (1.9)	8.6 (2.8)^a^	5.7 (1.6)	8.2 (2.7)^a^
Insulin (pmol/L)	59.3 (38.7)	132.4 (65.9)	222.0 (147.9)^a^	56.5 (24.2)	218.9 (118.7)^a^
HOMA‐IR	1.9 (1.4)	4.5 (2.5)	8.0 (6.4)^a^	1.8 (0.8)	7.8 (5.1)^a^
LH (IU/L)	6.9 (5.3)	8.2 (4.6)	7.6 (4.1)	6.3 (4.5)	8.3 (4.0)
FSH (IU/L)	5.4 (1.6)	5.4 (1.5)	5.5 (1.9)	5.0 (1.8)	5.8 (1.6)^c^
LH:FSH	1.2 (0.7)	1.5 (0.7)	1.4 (0.7)	1.3 (0.8)	1.4 (0.6)
E_2_ (pmol/L)	159.3 (86.4)	159.5 (55.5)	177.9 (66.5)	168.0 (84.1)	164.4 (59.3)
P (nmol/L)	1.9 (0.8)	2.5 (1.9)	2.1 (1.9)	2.8 (1.8)	2.7 (0.5)
17‐OHP (nmol/L)	1.9 (1.4)	1.5 (0.5)	1.4 (1.5)	1.5 (1.1)	1.6 (1.4)
A_4_(nmol/L)	5.5 (2.7)	6.2 (3.8)	4.7 (2.5)	4.8 (2.6)	5.5 (3.0)
T (nmol/L)	1.2 (0.5)	1.4 (0.6)	1.4 (0.7)	1.2 (0.5)	1.5 (0.7)
DHEAS (µmol/L)	6.3 (1.9)	8.3 (3.4)	6.1 (2.4)^b^	6.9 (2.7)	6.9 (2.9)
TC (mmol/L)	4.9 (0.8)	4.9 (0.8)	4.9 (1.1)	4.7 (0.9)	5.0 (0.9)
LDL‐C (mmol/L)	2.7 (0.7)	3.0 (0.8)	2.9 (0.8)	2.8 (0.8)	3.0 (0.8)
HDL‐C (mmol/L)	1.7 (0.6)	1.4 (0.7)	1.2 (0.2)^b^	1.5 (0.4)	1.3 (0.7)
TG (mmol/L)	1.0 (0.6)	1.5 (0.9)	1.6 (0.6)^c^	0.9 (0.4)	1.8 (0.7)^a^
TC:HDL‐C	3.1 (0.9)	4.0 (1.2)	4.1 (0.9)^b^	3.3 (0.8)	4.3 (1.1)^a^
Dietary intake					
Energy (kcal)	1,713 (1,380−1,943)	1,853 (1,628−1,979)	1,783 (1,512−1,928)	1,792 (1,346−1,976)	1,858 (1,660−1,992)
Steps (/day)	6,813 (5,138−11,360)	6,796 (4,902−9,402)	5,967 (4,605−8,123)	5,985 (4,930−8,337)	6,167 (4,664−8,297)
Protein (%)	16.7 (14.1−18.2)	16.7 (14.4−20.0)	17.6 (14.6−21.0)	16.7 (14.1−19.2)	17.4 (14.4−20.6)
Carbohydrate (%)	48.3 (41.1−53.9)	46.7 (43.6−50.6)	45.2 (41.4−48.2)	45.5 (42.1−51.8)	46.3 (43.3−50.3)
Fat (%)	34.9 (32.0−41.2)	36.0 (32.3−38.0)	36.0 (33.0−42.2)	36.1 (34.1−37.9)	35.1 (30.0−38.8)
Fiber (g)					
Crude	20.9 (17.1−26.5)	20.0 (15.1−23.8)	17.5 (15.7−22.1)	22.2 (15.6−26.5)	17.9 (16.0−22.6)
Adjusted	22.1 (18.5−26.2)	19.3 (15.9−23.2)	18.2 (14.4−23.6)	22.1 (17.6−26.2)	18.6 (14.0−23.4)^c^
Glycemic load					
Crude	69.3 (55.3−93.1)	93.4 (59.6−108.4)	84.6 (63.7−105.8)	69.3 (56.3−96.0)	93.2 (78.0−111.5)^b^
Adjusted	83.6 (67.0−96.3)	82.7 (60.8−104.3)	94.7 (68.4−108.2)	83.5 (61.4−95.9)	93.5 (73.6−109.4)^c^
Vitamin A (IU)					
Crude	7,883.0 (2,365.1−9,279.1)	6,985.76 (3,755.7−11,350.2)	3,055.8 (1,997.6−7,061.2)	6,863.28 (3,070.3−11,407.9)	4,737.14 (2,089.9−7,281.7)
Adjusted	7,807.5 (2,838.7−9,618.4)	6,979.9 (3,824.4−11,419.1)	3,296.1 (1,877.6−6,869.6)	7,162.02 (3,214.3−11,872.4)	4,433.22 (2,267.26−7,277.4)
Vitamin C (mg)					
Crude	103.7 (68.8−177.9)	107.8 (78.5−150.8)	63.8 (40.4−108.7)	100.4 (67.9−120.9)	91.33 (52.7−124.8)
Adjusted	103.2 (73.2−170.0)	106.1 (76.8−153.6)	66.8 (41.4−111.4)	98.9 (68.4−119.8)	87.8 (51.5−131.4)
Vitamin D (mcg)					
Crude	2.17 (1.18−4.71)	2.74 (1.61−5.15)	2.76 (1.77−3.88)	2.51 (1.21−4.71)	2.88 (1.58−4.17)
Adjusted	2.29 (1.43−4.00)	2.85 (1.68−5.38)	2.97 (1.63−3.89)	2.38 (1.63−4.46)	2.93 (1.63−4.11
Folate (mcg)					
Crude	227.2 (155.1−421.7)	264.7 (172.3−363.9)	268.8 (182.0−300.6)	237.8 (158.7−365.3)	267.9 (167.0−295.7)
Adjusted	242.6 (167.6−404.8)	266.2 (174.6−358.7)	254.9 (185.7−309.5)	231.5 (192.5−355.3)	252.8 (164.8−301.7)
Calcium (mg)					
Crude	710.8 (455.3−818.5)	552.6 (470.1−771.0)	580.6 (345.7−782.4)	687.5 (431.5−849.7)	569.5 (406.0−796.1)
Adjusted	748.6 (593.2−818.6)	576.3 (420.8−747.4)	616.1 (436.8−759.6)	686.6 (530.6−845.5)	604.2 (414.3−708.8)
Iron (mg)					
Crude	11.4 (9.0−14.8)	11.6 (9.5−14.3)	11.6 (8.7−13.1)	10.78 (8.88−14.3)	11.65 (9.65−14.0)
Adjusted	10.8 (9.5−14.7)	11.5 (9.4−13.0)	11.8 (8.7−13.1)	10.67 (9.35−13.83)	11.47 (9.03−13.13)
Sodium (mg)					
Crude	2,115.1 (1,886.9−2,932.5)	2,108.9 (1,379.3−2,824.5)	2,374.3 (1,601.6−3,092.2)	2,050.34 (1,522.4−2,710.3)	2,342.32 (1,590.0−3,553.4)
Adjusted	2,195.5 (1,889.4−2,915.8)	2,024.6 (1,607.3−2,480.1)	2,396.4 (1,888.1−2,972.2)	2,126.65 (1,691.7−2,508.1)	2,388.13 (1,612.6−3,116.0)
Zinc (mg)					
Crude	8.12 (6.06−10.0)	8.27 (6.04−10.56)	7.32 (5.30−10.24)	7.65 (5.55−9.15)	8.28 (5.97−11.16)
Adjusted	7.51 (6.23−9.14)	8.36 (7.30−10.5)	7.51 (5.28−10.33)	7.93 (6.37−10.43)	7.94 (6.78−10.31)
Magnesium (mg)					
Crude	251.0 (157.7−385.2)	241.5 (164.9−332.2)	200.4 (161.8−260.3)	240.7 (147.4−398.4)	212.3 (156.7−289.0)
Adjusted	247.0 (201.4−359.8)	243.5 (188.1−340.8)	210.5 (156.2−275.1)	264.5 (195.1−348.1)	208.4 (153.1−278.5)^c^
Cholesterol (mg)					
Crude	248.4 (120.8−349.7)	220.3 (173.6−352.5)	416.4 (225.4−568.3)	228.2 (171.2−368.6)	300.6 (183.4−507.0)
Adjusted	261.9 (126.4−350.3)	214.9 (183.5−368.9)	432.9 (221.2−559.0)	213.45 (178.8−374.9)	302.29 (178.8−504.1)

Note

Statistical significance where “a” denotes *p* ≤ 0.001, “b” denotes *p* ≤ 0.01, and “c” denotes *p* < 0.05. Values are expressed as mean (*SD*) or median (interquartile range). Fiber, glycemic load, and all micronutrients are presented in crude and adjusted intakes (refer to Table 1 note). BMI is categorized as normal (between 18.5 and 24.9 kg/m^2^), overweight (between 25 and 29.9 kg/m^2^), and obese (over 30 kg/m^2^). A HOMA‐IR of 3.8 or higher defines insulin resistance. Statistical tests were performed comparing anthropometric characteristics and dietary intake among women in normal, overweight, and obese BMI categories. In addition, statistical tests were performed comparing anthropometric characteristics and dietary intakes between women with insulin resistance and women without insulin resistance.

A_4_: androstenedione; DHEAS: dehydroepiandrosterone sulfate; FBG: fasting blood glucose; HDL‐C: high‐density lipoprotein cholesterol; HOMA‐IR: homeostasis model assessment of insulin resistance; LDL‐C: low‐density lipoprotein cholesterol; TC: total cholesterol; TG: triglycerides; 2 hr gluc: 2 hr glucose level following 75 g oral glucose tolerance test; 17‐OHP 17‐hydroxyprogesterone.

**Figure 1:**
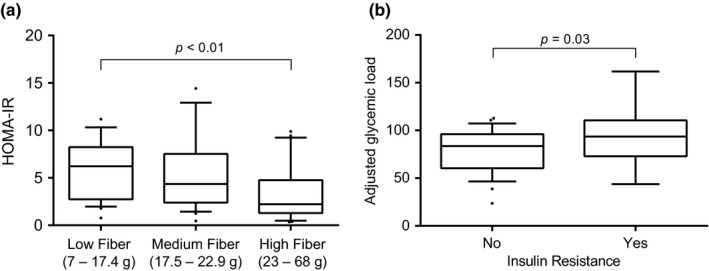
Insulin resistance and dietary intake. (a), HOMA‐IR of women with PCOS categorized by tertile of fiber intake (energy adjusted). (b), Glycemic load intake (energy adjusted) in women with PCOS categorized by insulin resistance diagnosis (based on HOMA‐IR ≥3.8). HOMA‐IR: homeostasis model assessment of insulin resistance

When women with PCOS were compared by BMI, no differences were found in dietary intake or activity level (Table 2). However, BMI was significantly greater in women with IR than those without (*p* < 0.001). Fiber intake was also significantly less in women with IR after adjusting for caloric intake (*p* < 0.05). When adjusted fiber intake was categorized by tertiles, HOMA‐IR differed significantly (*p* = 0.01), and women with the least fiber intake had significantly higher HOMA‐IR than women with the greatest fiber intake (*p* < 0.01, Figure 1). Furthermore, fiber intake was negatively correlated with HOMA‐IR (*ρ* = −0.35, *p* < 0.005), fasting insulin (*ρ* = −0.37, *p* < 0.005), 2 hr glucose level (*ρ* = −0.23, *p* < 0.05), triglycerides (*ρ* = −0.27, *p* = 0.02), total cholesterol/HDL‐C ratio (*ρ* = −0.29, *p* < 0.01), and positively correlated with HDL‐C (*ρ* = 0.28, *p* = 0.01). Fiber intake was not correlated with fasting blood glucose (FBG), total cholesterol, or LDL‐C.

Glycemic load was significantly greater for women with IR, both before (*p* < 0.01) and after (*p* = 0.03) adjusting for caloric intake (Table 2, Figure 1). Glycemic load, however, was not correlated with other metabolic or lipid parameters.

The effects of adjusted fiber intake, adjusted glycemic load, BMI, age, and PCOS phenotype on HOMA‐IR were further assessed by multiple linear regression analysis. Fiber intake and BMI were independent predictors of HOMA‐IR explaining 54.0% of the variance in the predictive model (*p* < 0.0001).

**Figure 2:**
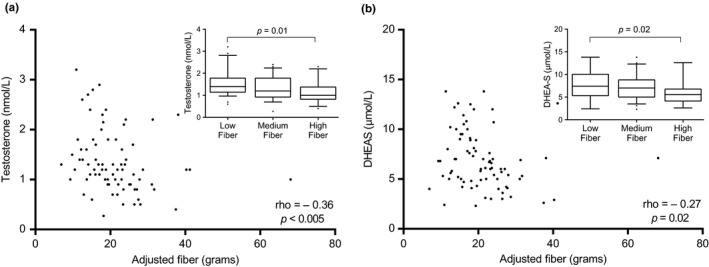
Fiber intake and androgen levels. Correlations between fiber intake (energy adjusted) in women with PCOS and (a) testosterone, and (b) DHEAS. Fiber intake (energy adjusted) categorized by tertiles and corresponding (a) testosterone and (b) DHEAS (low fiber = 6.9–17.4 g, medium fiber = 17.5–22.9 g, high fiber = 23.0–68.0 g). DHEAS: dehydroepiandrosterone sulfate

When the dietary intakes of women with PCOS were compared by phenotype (HA vs. Non‐HA), there were no significant differences in total caloric intake, activity, or macronutrient intake. However, fiber intake was negatively correlated with testosterone (*ρ* = −0.36, *p* < 0.005) and DHEAS (*ρ* = −0.27, *p* = 0.02; Figure 2). There were no correlations between androstenedione and any dietary components. When fiber intakes were grouped in tertiles, women with lower fiber intake had significantly higher testosterone (*p* = 0.01) and DHEAS (*p* = 0.02) levels than those with higher fiber intake (Figure 2).

In women with PCOS and insulin resistance (as assessed by HOMA), magnesium intake was decreased, after adjusting for caloric intake in comparison to women with PCOS without insulin resistance (208.4 vs. 264.5 mg, *p* = 0.04; Table 2). Insulin resistance was negatively correlated with magnesium intake (*ρ* = −0.27, *p = *0.02), but positively correlated with cholesterol intake (*ρ* = 0.26, *p* = 0.03). Magnesium intake was also negatively correlated with C‐reactive protein (*ρ* = −0.39, *p* < 0.01), and testosterone (*ρ* = −0.31, *p < *0.01), but positively correlated with HDL cholesterol (*ρ* = 0.28, *p = *0.02). None of the vitamin and mineral intakes assessed were related to BMI, waist‐to‐hip ratio, or PCOS phenotype.

## 4 DISCUSSION

Our study indicated that, despite significant differences in BMI and WHR, overall caloric intake and physical activity did not differ between women with and without PCOS, as have been observed in previous studies conducted in North America (Douglas et al., 2006; Wright et al., 2004). These findings demonstrate that obese women with PCOS are not in an energy surplus state supporting previous studies that suggest women with PCOS could indeed have an altered metabolism contributing to their obesity and IR (Georgopoulos et al., 2009; Huijgen et al., 2015; Robinson et al., 1992).

This is the first study to identify low fiber intake as a significant factor in IR in women with PCOS; women with IR (by HOMA‐IR score) consumed less fiber than women without IR (Table 2, Figure 1); and fiber intake was an independent predictor of HOMA‐IR. Other metabolic markers were also inversely associated with fiber intake as in the 2 hr glucose, fasting insulin, triglyceride levels, total cholesterol/HDL‐C ratio, and directly associated with HDL‐C. Although others have reported that dietary composition, including fiber intake, was not associated with IR in their PCOS cohorts, these previous studies had smaller sample sizes than ours (Douglas et al., 2006; Toscani, Mario, Radavelli‐Bagatini, & Spritzer, 2011). More importantly, fiber intake was not adjusted for total calories consumed as in our study. Similarly, while it was found that neither caloric nor macronutrient intake were associated with IR in women with PCOS, the use of 24‐hr dietary recall for only 1 day had a greater likelihood for error due to reliance on memory compared to a food record (Toscani et al., 2011). Furthermore, by obtaining 3 days of data, we accounted for day‐to‐day variability, without compromising participants’ attention to detail (which may occur with more than 4 days of records; Yang et al., 2010). Lastly, we found that fiber intake was significantly reduced in women with PCOS when compared to women without PCOS which is in agreement with findings by Wild et al., but in contrast to those of Moran et al. (Moran et al., 2013; Wild et al., 1985). Although Moran et al. performed a large, longitudinal study, one major limitation was that PCOS diagnoses were self‐reported, and therefore, the group with PCOS might have been underrepresented especially since PCOS could be undiagnosed in up to 70% of women (March et al., 2010; Moran et al., 2013).

In the general population, dietary fiber intake has been inversely associated with type 2 diabetes and cardiovascular disease, two conditions that share similar risk factors of metabolic syndrome as seen in PCOS (Bo et al., 2006; Threapleton et al., 2013; Weickert & Pfeiffer, 2008; Yao et al., 2014). Dietary fiber can help regulate blood glucose by slowing its absorption into the circulation thereby improving glucose tolerance. Soluble fiber lowers the postprandial glucose response while insoluble fiber increases insulin sensitivity (Weickert & Pfeiffer, 2008). The consumption of fiber has also been shown to help manage weight potentially through increased postprandial satiety resulting in reduced overall caloric consumption (Weickert & Pfeiffer, 2008). Indeed, women who consumed less dietary fiber had been shown to gain more weight over time (Liu et al., 2003). Even in populations with normal BMIs, reduced fiber intake has been associated with type 2 diabetes and metabolic syndrome (Bo et al., 2006). The daily recommended fiber intake for Canadian women is 25 g a day, but in our study, women with PCOS consumed only an average of 19.6 g per day. This small difference of 5–10 g of dietary soluble fiber daily has been shown to reduce LDL cholesterol by 5% (Brown, Rosner, Willett, & Sacks, 1999).

Women with PCOS have been reported to consume greater glycemic loads than women without PCOS (Douglas et al., 2006; Shishehgar et al., 2016). However, we found higher glycemic loads in PCOS only in those with IR (Table 2) similar to findings reported by Graff et al. (2013). Intake of carbohydrates with a low glycemic index reduces the rate of glucose absorption. In turn, duodenal enterocyte hormone secretion of incretins stimulates insulin secretion to lower glucose levels. Reduction in the glucose load over an extended period suppresses free fatty acid levels and improves insulin sensitivity and glucose levels (Jenkins et al., 2002). In non‐PCOS populations, meta‐analyses of randomized controlled trials have shown that low‐glycemic diets can reduce fasting insulin levels, pro‐inflammatory markers, total and LDL cholesterol (Brand‐Miller, Holt, Pawlak, & McMillan, 2002; Goff, Cowland, Hooper, & Frost, 2013; Schwingshackl & Hoffmann, 2013). Meals with increased fiber can also alter the glycemic response and reduce glucose absorption by either hindering glucose absorption in the small intestine, and/or inhibiting α‐amylase action (Ou, Kwok, Li, & Fu, 2001). Therefore, we suggest both glycemic load and fiber be reported in studies as they are interrelated. Examining total glycemic load alone without a fiber analysis would fail to account for the effect of mixed meals (Bonora et al., 2000). Our results support implementing high‐fiber, low‐glycemic meals in the management of IR in patients with PCOS (Barr, Reeves, Sharp, & Jeanes, 2013; Marsh et al., 2010).

While serum magnesium deficiencies have been previously reported, this is the first study to find associations between habitual lower dietary magnesium intake and hormonal/metabolic outcomes in women with PCOS. Further, our results on vitamin A and cholesterol intakes are consistent with the biological roles of these micronutrients and emphasize the equal importance of refining dietary components in advising lifestyle changes in PCOS. Vitamin A has been implicated in animal studies as essential for fetal pancreatic beta cell growth and development. Its deficiency is associated with diabetes in these animals (Matthews, Rhoten, Driscoll, & Chertow, 2004). A more recent study further implicates an important role for a vitamin A metabolite, all‐trans retinoic acid, on human pancreatic beta islet cell function and thereby insulin output via the GPRC5C receptor (Amisten et al., 2017).

Polycystic ovary syndrome is a spectrum disorder resulting in phenotypic differences. Hyperandrogenism can increase the severity and associated risks of PCOS, and also contribute to anxiety, low self‐esteem, poor body image, and loss of female identity (Bazarganipour et al., 2013; Kitzinger & Willmott, 1982; Livadas et al., 2011). Our analysis comparing women with HA PCOS to Non‐HA PCOS did not show any differences in dietary intake, but when fiber intake was categorized by tertiles, testosterone and DHEAS were increased in those who consumed less fiber. It is known that higher BMI, as well as IR with compensatory hyperinsulinemia (IR/HI), can exacerbate hyperandrogenism (Barbieri & Ryan, 1983; Lim et al., 2012). While we did not find an association between BMI and fiber, our findings were consistent with the well‐documented association between IR/HI and hyperandrogenism and identified a potential dietary target to improve IR/HI and hyperandrogenism. Katcher et al. demonstrated differences in acute postprandial testosterone and DHEAS levels were dependent on meal composition (a high‐fiber, low fat meal was compared to a high fat, low fiber meal; Katcher et al., 2009). Our study further demonstrated that regular dietary fiber intake over a long time period correlates not only with IR/HI, but with testosterone and DHEAS levels. While our study utilized a standard androgen profile, recent studies have shown that adrenal 11‐oxygenated androgens are substantial contributors to the total circulating androgen pool in PCOS and correlate with IR (O'Reilly et al., 2017). Nonetheless, our results indicate that increasing fiber is an important dietary target in the management of hyperandrogenic PCOS.

A main strength of our study was our adjustment of nutrient intakes, using the residual method (see Table 1 notes), to control for total amount of food consumed. In epidemiologic studies, potential associations between the prevalence of disease and specific nutrient intakes can be overlooked if variations in total energy intake are not adjusted (Willett et al., 1997). Other strengths include our analysis of dietary differences between PCOS phenotypes and the use of an objective method (a pedometer) to evaluate physical activity, both recommended in a recent literature review (Lin & Lujan, 2014). Additionally, PCOS as a diagnosis was strictly defined according to the Rotterdam criteria and assessed through consistent methods by one reproductive endocrinologist (APC). Some of these factors may contribute to varied findings from the nutritional studies available on PCOS. Finally, our study is the first to assess dietary intake and physical activity in Canadian women with PCOS (Lujan, Chizen, & Pierson, 2008).

While women with PCOS were younger, the difference of 5 years in the reproductive age group would be expected to have little impact on metabolic health or dietary intake. Underreporting can be a limitation when assessing dietary intake through self‐report methods, especially in groups more likely to underreport (ex. those with higher BMI; Gemming, Jiang, Swinburn, Utter, & Mhurchu, 2014). However, removing under‐reporters, according to the Goldberg cutoff method, did not affect the statistical significance found (Black, 2000; Schofield, 1985). Miscommunication of portion size is a common limitation in self‐report food records but this was addressed by encouraging the use of measuring tools, the addition of food photographs, and providing participants with a list of common objects to compare to their portion sizes. Finally, our findings apply to women with PCOS whose primary reason for seeking medical care is infertility and may not be generalizable to all women with PCOS.

## 5 CONCLUSIONS

In conclusion, we found that women with PCOS and obesity were not in a caloric surplus state. However, dietary components, specifically low fiber, low magnesium, low vitamin A and high glycemic load, may contribute to IR/HI and obesity. In addition, low fiber intake may contribute to hyperandrogenemia. Future randomized controlled trials are required to determine the benefit of high‐fiber, low‐glycemic diets in improving glucose tolerance, and preventing metabolic complications in women with PCOS.

## CONFLICT OF INTEREST

The authors have no conflict of interests to declare.

## ETHICAL STATEMENTS

This study was approved by the University of British Columbia's Children's and Women's Research Ethics Board (registration number H13‐03441), and written informed consent was obtained from all participants. The corresponding author (APC) affirms that this manuscript is an honest, accurate, and transparent account of the study being reported. The reporting of this work is compliant with STROBE guidelines. APC affirms that no important aspects of the study have been omitted and that any discrepancies from the study as planned have been explained.
